# Association Between Low Anterior Resection Syndrome and Lower Urinary Tract Symptom Severity After Restorative Rectal Cancer Surgery

**DOI:** 10.3390/medsci14060682

**Published:** 2026-10-19

**Authors:** Sorinel Lunca, Stefan Morarasu, Cristian Ene Roata, Gabriel Mihail Dimofte

**Affiliations:** 1 Grigore T. Popa University of Medicine and Pharmacy, 700115 Iasi, Romania; sorinel.lunca@umfiasi.ro (S.L.); cristian.roata@umfiasi.ro (C.E.R.); mihail.dimofte@umfiasi.ro (G.M.D.); 2 2nd Department of Surgical Oncology, Regional Institute of Oncology, 700483 Iasi, Romania

**Keywords:** low anterior resection syndrome, LARS score, lower urinary tract symptoms, International Prostate Symptom Score, rectal cancer, pelvic functional symptoms, ordinal regression

## Abstract

**Background:** Low anterior resection syndrome (LARS) and lower urinary tract symptoms (LUTS) are common after restorative rectal cancer surgery, but whether their postoperative severity is associated after accounting for relevant clinical, anatomical, and treatment-related factors remains unclear. **Methods:** We conducted a retrospective, single-centre cohort study of patients treated for rectal cancer at the Regional Institute of Oncology, Iași, Romania, between 2013 and 2024. Patients were eligible if they underwent sphincter-preserving rectal resection, achieved restoration of bowel continuity, and completed the LARS score and International Prostate Symptom Score (IPSS) during the same postoperative telephone assessment. The primary analysis used proportional-odds ordinal logistic regression to evaluate the association between LARS and IPSS categories after adjustment for neoadjuvant radiotherapy, chemotherapy, extent of mesorectal excision, MRI-measured distance from the tumour to the anal verge, age, sex, body mass index, diabetes, and smoking. Multiple sensitivity and model-diagnostic analyses were performed to assess the robustness of the primary association. **Results:** Of 404 patients managed during the study period, 190 were included in the analytic cohort. Major LARS was present in 53 patients (27.9%), and moderate-to-severe LUTS in 84 (44.2%). LARS score and IPSS were modestly correlated (Pearson r = 0.225, *p* = 0.0018; Spearman ρ = 0.237, *p* = 0.0010). Moderate-to-severe LUTS occurred in 31 of 53 patients with major LARS (58.5%) and in 53 of 137 with no or minor LARS (38.7%; *p* = 0.021). In the primary adjusted model, each one-category increase in LARS severity was associated with greater IPSS severity (OR 1.75, 95% CI 1.25–2.46, *p* = 0.0012), and addition of LARS significantly improved model fit (likelihood-ratio χ^2^ = 10.85, *p* = 0.0010). Major LARS was associated with approximately threefold higher odds of greater IPSS severity compared with no LARS (OR 3.05, 95% CI 1.54–6.04, *p* = 0.0014). Diabetes was also associated with greater IPSS severity after multivariable adjustment (OR 3.32, 95% CI 1.44–7.67, *p* = 0.0049). Results remained materially unchanged across sensitivity analyses. Item-level correlations were weak, and none remained significant after Bonferroni correction. **Conclusions:** Greater postoperative LARS severity was associated with greater contemporaneously measured LUTS severity after multivariable adjustment. This association was consistent across multiple correlated sensitivity analyses but does not establish temporal direction or a shared causal mechanism. Patients with major LARS may therefore warrant concurrent assessment for urinary symptoms during postoperative and survivorship follow-up.

## 1. Introduction

Improvements in multimodal treatment, total mesorectal excision, and sphincter-preserving surgery have increased the number of patients living after rectal cancer treatment with restored intestinal continuity. As oncological outcomes have improved, long-term function and quality of life have become increasingly important components of treatment evaluation. Avoidance of a permanent stoma, however, does not necessarily imply preservation of normal bowel function. Many patients experience faecal or flatus incontinence, urgency, clustering of bowel movements, increased stool frequency, and difficulty with evacuation after restorative rectal resection. These symptoms are collectively described as low anterior resection syndrome (LARS), defined by international consensus as disordered bowel function after rectal resection that adversely affects quality of life [1,2,3].

The LARS score is a concise, validated patient-reported instrument comprising five weighted items—flatus incontinence, liquid-stool leakage, bowel frequency, clustering, and urgency—and classifies patients as having no, minor, or major LARS [1]. Meta-analyses have estimated the prevalence of major LARS after restorative rectal cancer surgery at approximately 40–44%, although reported rates vary according to tumour height, neoadjuvant treatment, surgical technique, anastomotic leakage, temporary diversion, follow-up duration, and patient selection [4,5]. Prospective assessments have also shown that symptom severity may improve during the first postoperative year, although substantial bowel dysfunction can persist after restoration of bowel continuity [6,7]. However, major-LARS-range symptoms have also been reported in individuals without rectal surgery, particularly among those aged 50–79 years and those with physical disease, underscoring that the score reflects symptom burden rather than a mechanism specific to rectal resection [8].

The pathophysiology of LARS is multifactorial. Loss of the native rectal reservoir reduces storage capacity and compliance, while creation of a neorectum may alter colonic motility, anorectal sensation, evacuation, and the rectoanal inhibitory reflex. Internal sphincter injury, autonomic denervation, low anastomotic level, temporary faecal diversion, anastomotic complications, and radiation-related fibrosis or neuromuscular injury may contribute in varying combinations [2,3,9]. Tumour height, neoadjuvant radiotherapy, total mesorectal excision, anastomotic leakage, and diverting ileostomy are among the most consistently reported clinical risk factors for major LARS [4,5,9,10,11]. These observations informed the development of the Pre-Operative LARS score, which estimates the risk of postoperative bowel dysfunction and supports preoperative counselling [12]. Nevertheless, substantial interindividual variation remains, suggesting that conventional clinical predictors do not fully capture postoperative functional susceptibility.

Lower urinary tract symptoms (LUTS) represent another important but less consistently assessed consequence of rectal cancer treatment. LUTS include storage symptoms such as frequency, urgency, and nocturia, as well as voiding symptoms such as weak urinary stream, intermittency, straining, and incomplete emptying. A recent systematic review and meta-analysis found that approximately one in five patients experienced urinary dysfunction more than one year after rectal cancer surgery, although estimates varied because of heterogeneous definitions, instruments, and assessment intervals [13]. Prospective cohort evidence also indicates that urinary symptoms may already be present before treatment and may follow heterogeneous postoperative trajectories, reinforcing the importance of baseline assessment [14,15]. Consequently, postoperative LUTS cannot be assumed to represent treatment-induced dysfunction without standardised preoperative assessment, and observed postoperative associations may partly reflect pre-existing symptom burden.

Pelvic autonomic nerve injury is an established potential mechanism of urinary dysfunction following rectal resection. Sympathetic fibres from the superior hypogastric plexus and hypogastric nerves, parasympathetic fibres from the pelvic splanchnic nerves, and their convergence within the inferior hypogastric plexus contribute to bladder storage, detrusor contraction, bladder-neck function, and coordinated voiding. These neural structures lie close to the vascular pedicles, mesorectal fascia, pelvic sidewalls, seminal vesicles, prostate, vagina, and pelvic floor and may be exposed to traction, thermal injury, partial division, or postoperative fibrosis during rectal mobilisation [16,17,18]. Early studies of autonomic nerve-preserving total mesorectal excision documented clinically important urinary and sexual dysfunction in both men and women [17]. Data from the Dutch TME trial subsequently suggested that postoperative urinary dysfunction was associated principally with surgical nerve injury rather than preoperative radiotherapy [19].

The close anatomical and functional relationships among the rectum, anal sphincters, urinary bladder, urethra, and pelvic floor provide a plausible basis for concurrent bowel and urinary symptoms. Injury to pelvic autonomic or somatic pathways may affect more than one pelvic organ, while altered pelvic floor coordination, afferent sensory processing, postoperative fibrosis, and broader susceptibility to functional symptoms may contribute to parallel symptom burdens even in the absence of a single identifiable neural lesion. In a cadaveric and clinical extension of the Dutch TME trial, Wallner et al. identified neural structures at risk during low pelvic dissection and reported an association between newly developed faecal and urinary incontinence [20]. In women after rectal cancer resection, bowel dysfunction has also been associated with urinary dysfunction and impaired sexual function, further suggesting that postoperative pelvic dysfunction may involve multiple functional domains [21,22]. These observations support concurrent assessment but do not establish that bowel and urinary symptoms necessarily share one causal mechanism.

Despite this anatomical and clinical overlap, bowel and urinary outcomes have commonly been investigated separately [9,13,23]. LARS research has focused predominantly on bowel symptom severity, risk prediction, quality of life, and treatment, whereas urinary studies have often concentrated on postoperative retention, catheter dependency, incontinence, sexual function, or comparisons between operative approaches. A recent cross-sectional study assessing both domains after sphincter-preserving rectal cancer surgery found that major LARS and moderate-to-severe LUTS were common and that increasing severity in either domain was associated with poorer quality of life [23]. Previous studies assessing both functional domains have primarily described symptom prevalence, coexistence, or quality-of-life effects, with limited adjustment for factors that may contribute to both bowel and urinary dysfunction. Whether graded LARS severity remains associated with graded LUTS burden after multivariable adjustment therefore remains insufficiently defined.

The International Prostate Symptom Score (IPSS), derived from the American Urological Association symptom index, is a seven-item patient-reported measure covering urinary frequency, urgency, nocturia, incomplete emptying, intermittency, weak stream, and straining [24]. Although originally developed for men with symptoms of benign prostatic enlargement, it has been used more broadly to quantify LUTS, including in women and mixed-sex surgical populations. Psychometric evaluation has supported its relevance for assessing LUTS in women, although patient characteristics may influence its measurement properties [25]. The IPSS should therefore be interpreted as a measure of overall urinary symptom burden rather than as a diagnostic test for postoperative autonomic nerve injury. Elevated scores may reflect benign prostatic enlargement, overactive bladder, diabetes, ageing, medication effects, pre-existing urological disease, pelvic surgical injury, or combinations of these factors [16,25,26].

The present study therefore aimed to evaluate the association between LARS severity and LUTS severity, measured by IPSS, in patients undergoing restorative rectal cancer surgery, with multivariable adjustment for relevant clinical and treatment-related factors. Secondary analyses examined the clinical co-occurrence and symptom-level relationship between LARS and LUTS and assessed the robustness of the association across alternative model specifications and clinically relevant subgroups.

## 2. Materials and Methods

### 2.1. Study Design and Setting

This retrospective, single-centre, single-department cohort study included patients with rectal cancer managed at the Regional Institute of Oncology (IRO), Iași, Romania, between 2013 and 2024 and incorporated a cross-sectional postoperative functional assessment. All patients underwent oncological evaluation, treatment, and follow-up at the study institution. Treatment decisions, including surgery alone or surgery combined with neoadjuvant and/or adjuvant therapy, were made by a multidisciplinary tumour board in accordance with contemporary clinical practice. This study was reported in accordance with the Strengthening the Reporting of Observational Studies in Epidemiology (STROBE) statement (Supplementary Table S1).

### 2.2. Study Population and Eligibility Criteria

The institutional cohort comprised 404 patients managed for rectal cancer during the study period. Patients were eligible for the present analysis if they underwent sphincter-preserving rectal resection, subsequently achieved restoration of bowel continuity, and completed both the Low Anterior Resection Syndrome (LARS) score and the International Prostate Symptom Score (IPSS) during the same postoperative telephone assessment.

**Figure 1:**
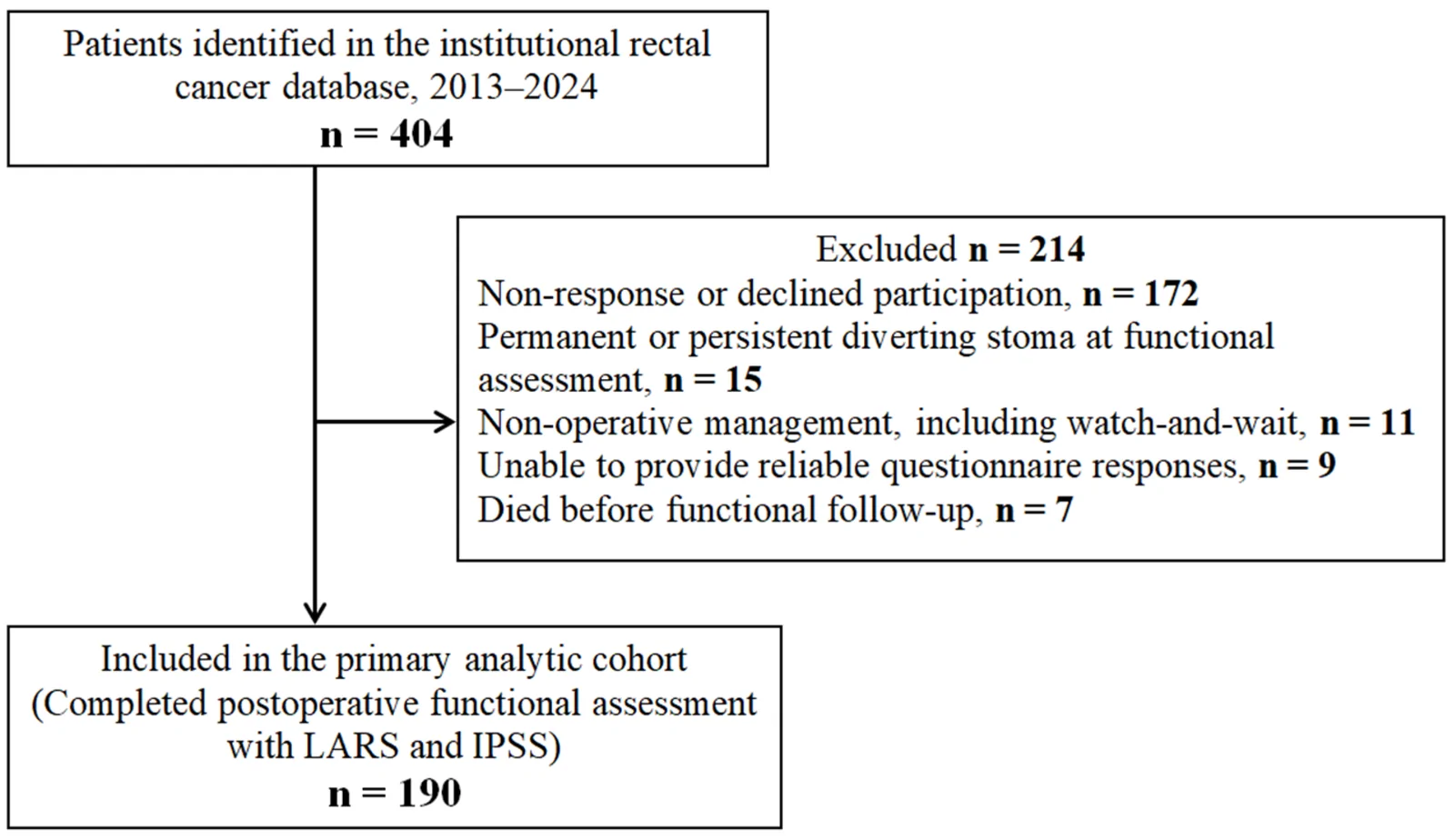
Flowchart of patient selection.

Patients were excluded if they had a permanent stoma or a persistent diverting stoma at the time of functional assessment (n = 15), died before functional follow-up (n = 7), did not respond or declined participation (n = 172), were unable to provide reliable questionnaire responses (n = 9), or were managed non-operatively, including by a watch-and-wait strategy (n = 11). Overall, 214 patients were excluded, leaving 190 patients in the final analytic cohort (Figure 1).

The primary analyses included all 190 patients. Item-level data were complete for 175 patients, follow-up from restoration of bowel continuity was available for 181, and protective ileostomy status was available for 182. The corresponding analytical subsets are shown in Supplementary Figure S1. No a priori sample-size calculation was performed; the study included all eligible patients with the required postoperative functional assessments available during the study period.

### 2.3. Functional Assessment

Postoperative bowel and lower urinary tract symptoms were assessed during the same structured telephone interview using the standard five-item LARS and the seven-item IPSS questionnaire [1,23]. Interviews were conducted by one of two trained assessors after restoration of bowel continuity.

Follow-up was calculated from restoration of bowel continuity, rectal resection in patients without diversion and ileostomy closure in those with a diverting ileostomy, to questionnaire administration.

The LARS score was classified as no LARS (0–20), minor LARS (21–29), or major LARS (30–42) [1]. IPSS was classified as no symptoms (0), mild symptoms (1–7), moderate symptoms (8–19), or severe symptoms (20–35) [23] and was interpreted as a measure of overall LUTS burden rather than as a diagnosis of a specific urological disorder.

### 2.4. Clinical, Tumour, and Treatment Variables

Demographic and clinical variables included age, sex, body mass index, diabetes, and smoking history. Smoking was classified as ever versus never smoking.

Treatment-related variables included neoadjuvant radiotherapy, perioperative chemotherapy, extent of mesorectal excision, and creation of a protective ileostomy. Mesorectal excision was classified as total or partial according to the operative record.

Tumour height was determined from preoperative pelvic magnetic resonance imaging. The distance from the inferior tumour margin to the anal verge was recorded as a continuous variable in millimetres. For categorical analyses, tumour position was classified as lower rectum (<5 cm from the anal verge), middle rectum (5–10 cm), or upper rectum (>10 cm).

Follow-up duration from restoration of bowel continuity to questionnaire assessment was evaluated in a sensitivity analysis rather than included in the primary model.

Additional variables examined in sensitivity analyses included a documented history of urinary symptoms before rectal surgery, anastomotic leakage/pelvic sepsis, anastomotic configuration, rectal stump length as the available measure related to anastomotic level, and postoperative medication for digestive or urinary symptoms. Pre-existing urinary symptoms were recorded as a binary historical variable; standardised preoperative IPSS measurements were not available. All patients received routine postoperative lifestyle advice during follow-up. No psychiatric disorders were documented in any of the patients included in the study. Anastomotic leakage/pelvic sepsis was available for the full cohort, anastomotic configuration for 182 patients, and rectal stump length, used as the available measure related to anastomotic level, was available in 110 patients.

### 2.5. Study Outcomes and Analytical Framework

The primary outcome was LUTS severity, represented by the ordered IPSS categories of none, mild, moderate, and severe. The primary exposure was LARS severity, represented by the ordered categories of no, minor, and major LARS. The primary analysis assessed the association between postoperative LARS and IPSS severity after adjustment for predefined demographic, clinical, anatomical, and treatment-related variables. Secondary analyses examined the correlation between continuous LARS score and IPSS, the prevalence of moderate-to-severe LUTS according to major-LARS status, and exploratory correlations between individual questionnaire items. Standardised preoperative LARS and IPSS measurements were not available; therefore, the principal analyses evaluated cross-sectional postoperative associations. A documented history of pre-existing urinary symptoms was examined separately in sensitivity analysis.

### 2.6. Statistical Analysis

Continuous variables were summarised as mean ± standard deviation or median with interquartile range and range, as appropriate. Categorical variables were reported as frequencies and percentages. Associations between continuous LARS score and IPSS were assessed using Pearson and Spearman correlation coefficients. The prevalence of moderate-to-severe LUTS in patients with major versus no or minor LARS was compared using the chi-square test with Yates’ continuity correction. Exploratory correlations between the five LARS and seven IPSS items were assessed using Spearman coefficients, with a Bonferroni-corrected significance threshold of *p* < 0.0014 for 35 comparisons.

Multivariable associations were examined using proportional-odds ordinal logistic regression. Model A used LARS category as the outcome, and Model B used IPSS category as the outcome. Both models included neoadjuvant radiotherapy, chemotherapy, extent of mesorectal excision, MRI-measured distance from the tumour to the anal verge, age, sex, body mass index, diabetes, and smoking. The primary Model C added LARS category as an ordered predictor to Model B. Models B and C were compared using a likelihood-ratio test. Age, body mass index, and tumour distance were standardised before modelling. Results were reported as odds ratios with 95% confidence intervals and *p*-values.

Sensitivity analyses evaluated alternative coding of tumour position and LARS severity, adjustment for follow-up duration and protective ileostomy, exclusion of patients assessed within 3 or 6 months of restoration of bowel continuity, continuous-score regression, interaction and stratification by sex, consolidation of moderate and severe IPSS categories, bootstrap estimation of the LARS coefficient, and potential separation at the severe-IPSS threshold. Additional sensitivity analyses examined pre-existing urinary symptoms, anastomotic leakage/pelvic sepsis, anastomotic configuration, rectal stump length, and postoperative digestive- and urinary-symptom medication. The proportional-odds assumption was assessed using the Brant test, and multicollinearity was evaluated using variance inflation factors. Models A, B, and C were fitted in the full analytic cohort (n = 190), as all variables required for these models were complete. Sensitivity analyses involving variables with missing data used the corresponding available-case subsets; no imputation was performed. All tests were two-sided, with *p* < 0.05 considered statistically significant except for the item-level analysis. Analyses were performed using Stata version 19 (StataCorp LLC, College Station, TX, USA).

### 2.7. Ethics

The Institution’s Ethics Committee waived the requirement for formal ethical approval, as this was a non-interventional, observational study involving anonymized data that posed no risk to participants. All study data were handled in accordance with institutional confidentiality and data-protection requirements. Prior to administering the questionnaire, verbal informed consent was obtained from each participant.

## 3. Results

### 3.1. Cohort Characteristics

A total of 190 patients were included. The mean age was 64.8 ± 9.9 years, 87 patients (45.8%) were women, and 31 (16.3%) had diabetes. Neoadjuvant radiotherapy was administered to 114 patients (60.0%), chemotherapy to 144 (75.8%), and total mesorectal excision was performed in 141 (74.2%). Protective ileostomy was created in 116 of 182 patients with available data (63.7%). Median follow-up from restoration of bowel continuity was 26.6 months (IQR 13.5–63.9; n = 181). Major LARS was present in 53 patients (27.9%), and moderate-to-severe LUTS in 84 (44.2%). Complete cohort characteristics are presented in Table 1.

### 3.2. Association Between LARS Score and IPSS and Clinical Co-Occurrence

**Figure 2:**
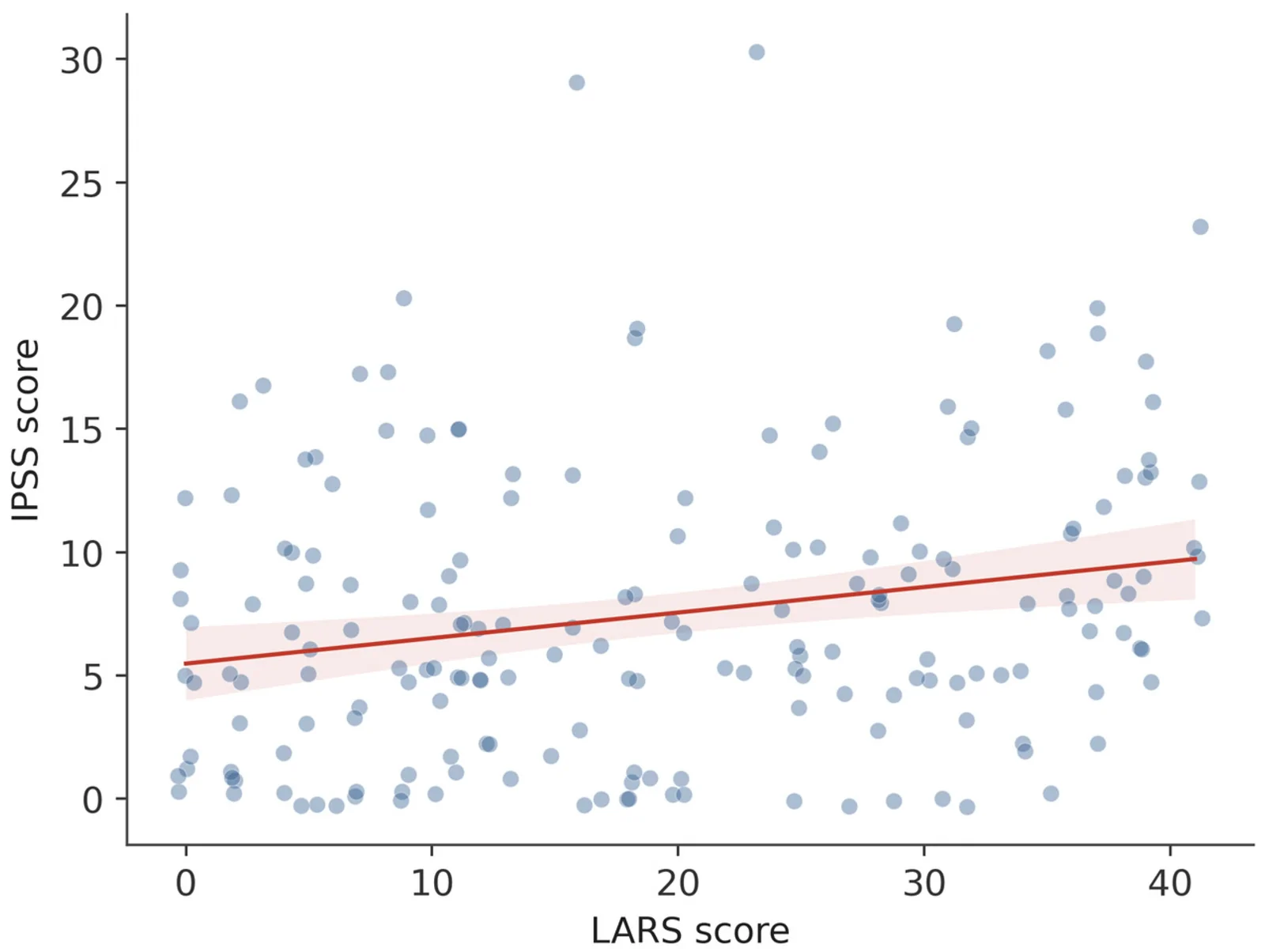
Association between LARS score and IPSS. The line represents the fitted linear regression with 95% confidence interval; points are jittered for visualisation only.

**Figure 3:**
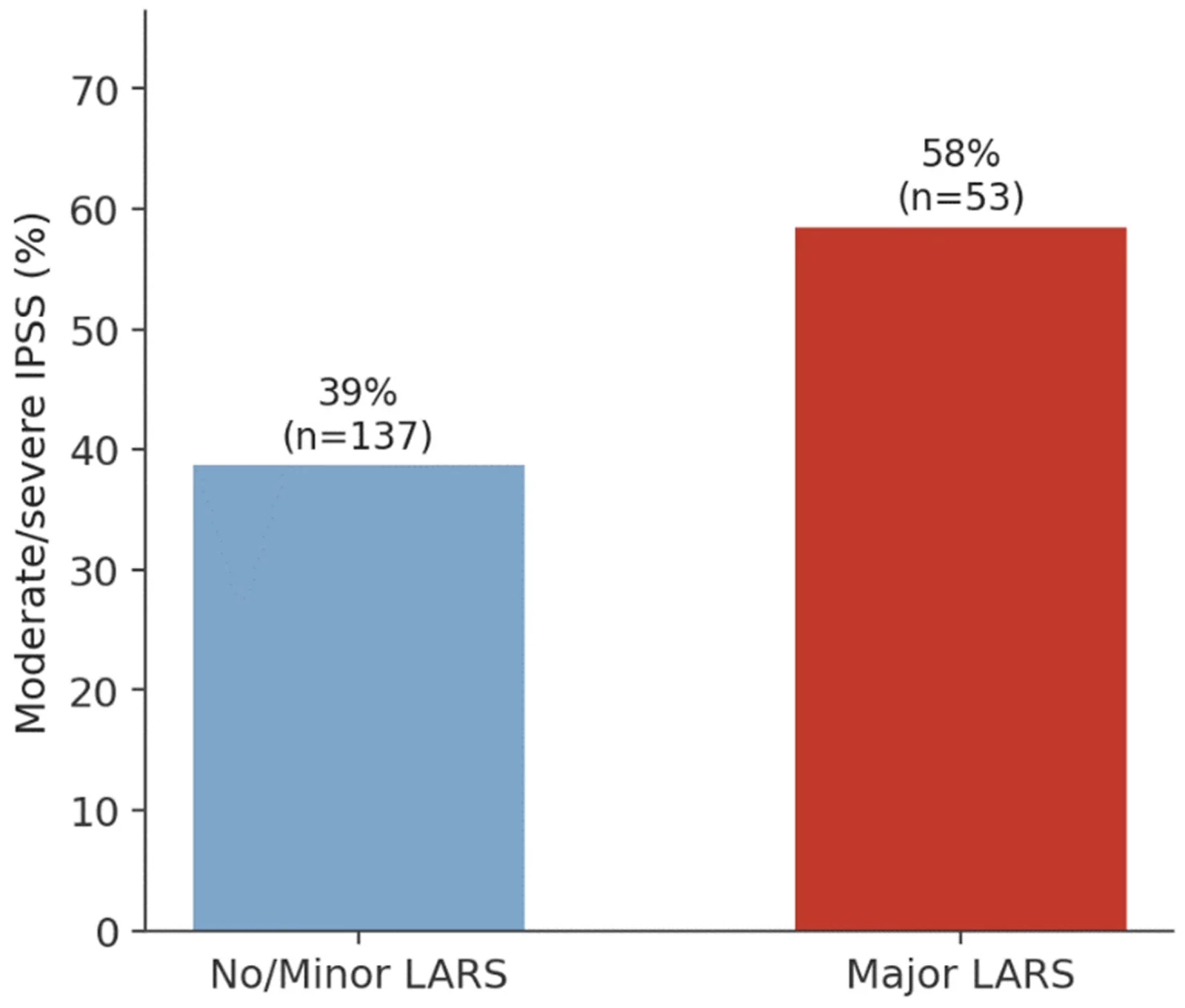
Prevalence of moderate-to-severe lower urinary tract symptoms according to major-LARS status.

LARS score and IPSS were modestly correlated (Pearson r = 0.225, *p* = 0.0018; Spearman ρ = 0.237, *p* = 0.0010; n = 190). Moderate-to-severe LUTS were present in 31 of 53 patients with major LARS (58.5%) and 53 of 137 patients with no or minor LARS (38.7%), an absolute difference of 19.8 percentage points (Yates-corrected χ^2^ = 5.30, *p* = 0.021). These associations are shown in Figure 2 and Figure 3.

### 3.3. Exploratory Item-Level Associations

**Figure 4:**
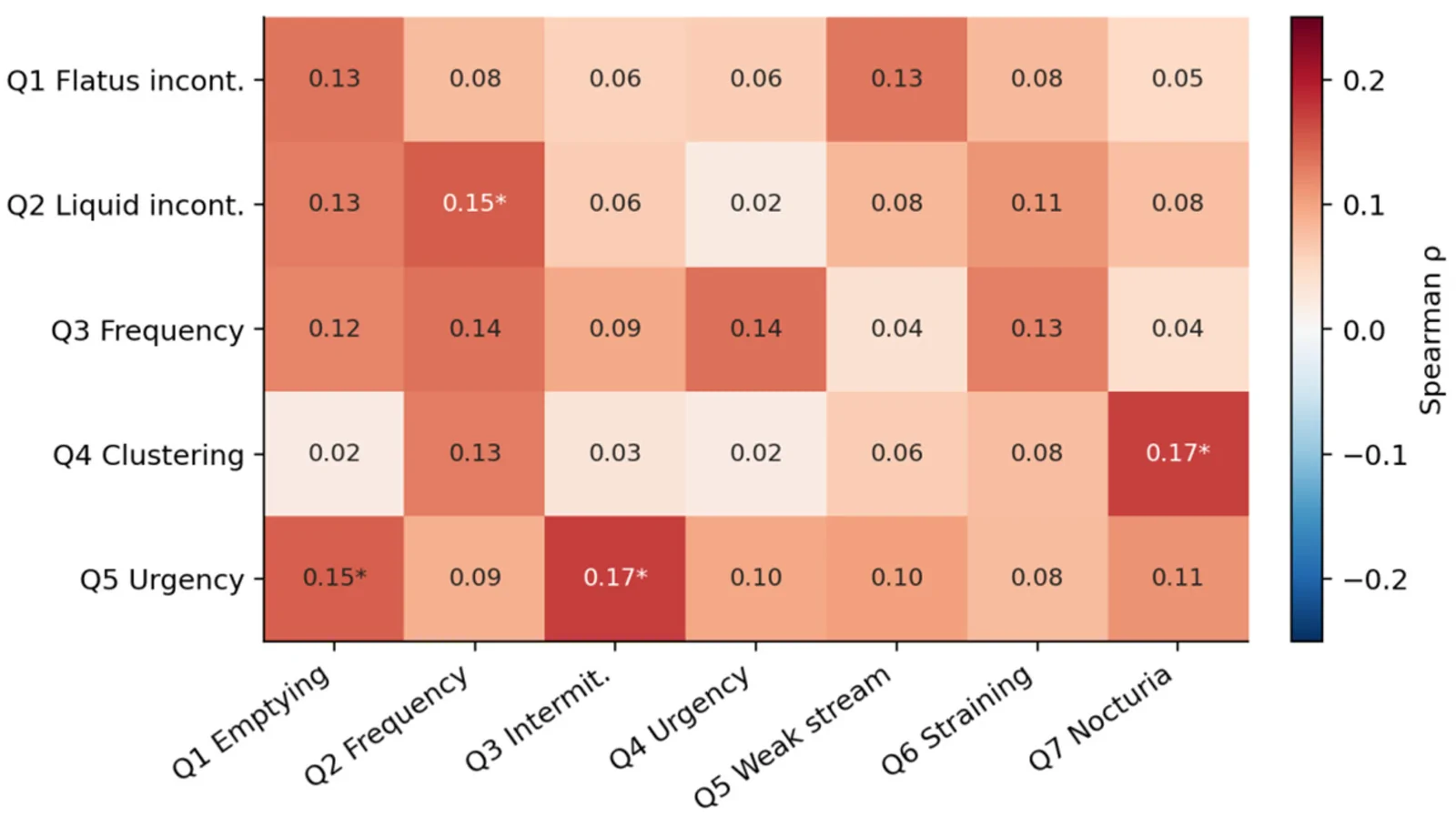
Heatmap of item-level correlations between LARS and IPSS symptoms. Values represent Spearman correlation coefficients; asterisks denote nominal *p* < 0.05.

Complete item-level data were available for 175 patients. All correlations between individual LARS and IPSS items were weak (|ρ| < 0.18). Four associations reached nominal *p* < 0.05, but none met the Bonferroni-corrected threshold of *p* < 0.0014 (Table 2; Figure 4).

### 3.4. Multivariable Ordinal Regression

All primary regression models were based on the full analytic cohort of 190 patients.

#### 3.4.1. Model A: Factors Associated with LARS Severity

**Figure 5:**
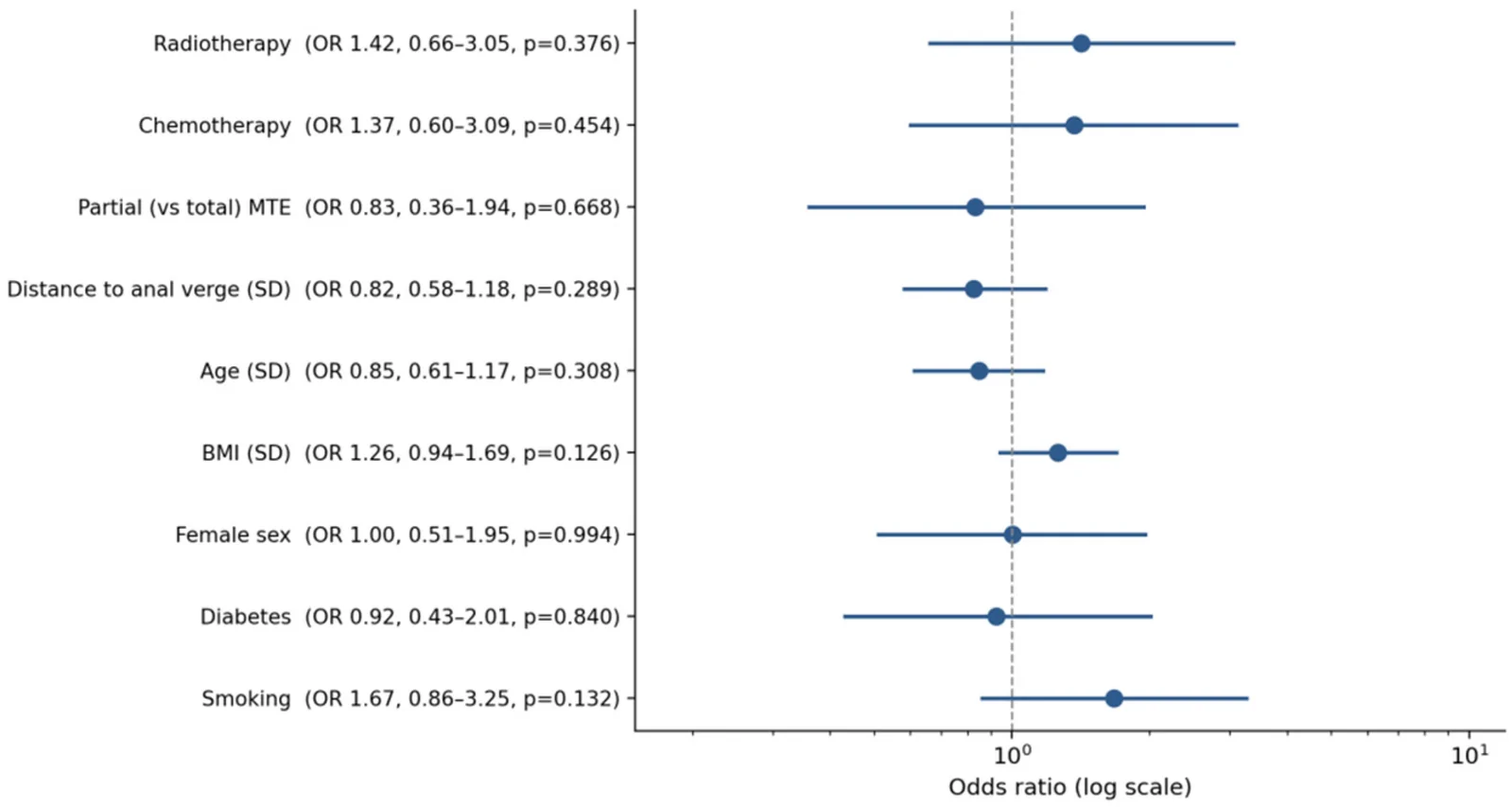
Forest plot of Model A estimates for LARS severity. Odds ratios are shown with 95% confidence intervals.

Model A examined associations between the predefined demographic, clinical, anatomical, and treatment-related variables and LARS category in the full cohort (n = 190). Model A did not significantly improve fit compared with the intercept-only model (likelihood-ratio χ^2^ = 16.61, df = 9, *p* = 0.055). None of the predefined covariates were significantly associated with LARS severity (Table 3; Figure 5).

#### 3.4.2. Model B: Factors Associated with IPSS Severity

**Figure 6:**
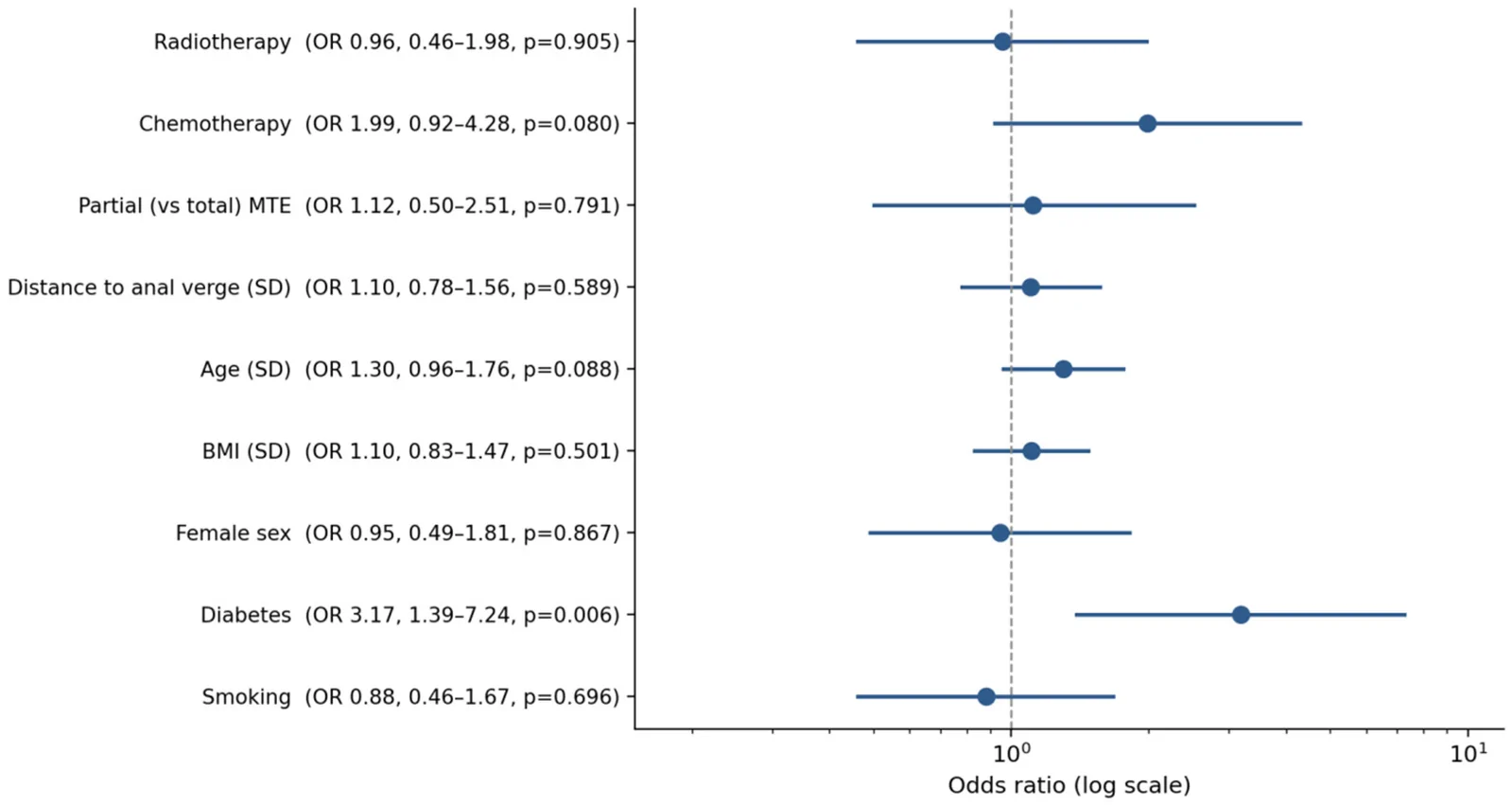
Forest plot of Model B estimates for IPSS severity. Odds ratios are shown with 95% confidence intervals.

Model B included the same covariates as Model A but used IPSS category as the outcome. Model B improved fit compared with the intercept-only model (likelihood-ratio χ^2^ = 17.91, df = 9, *p* = 0.036). Diabetes was associated with greater IPSS severity (OR 3.17, 95% CI 1.39–7.24, *p* = 0.006); no other covariate reached statistical significance (Table 4; Figure 6).

#### 3.4.3. Model C: Primary Model Including LARS Severity

**Figure 7:**
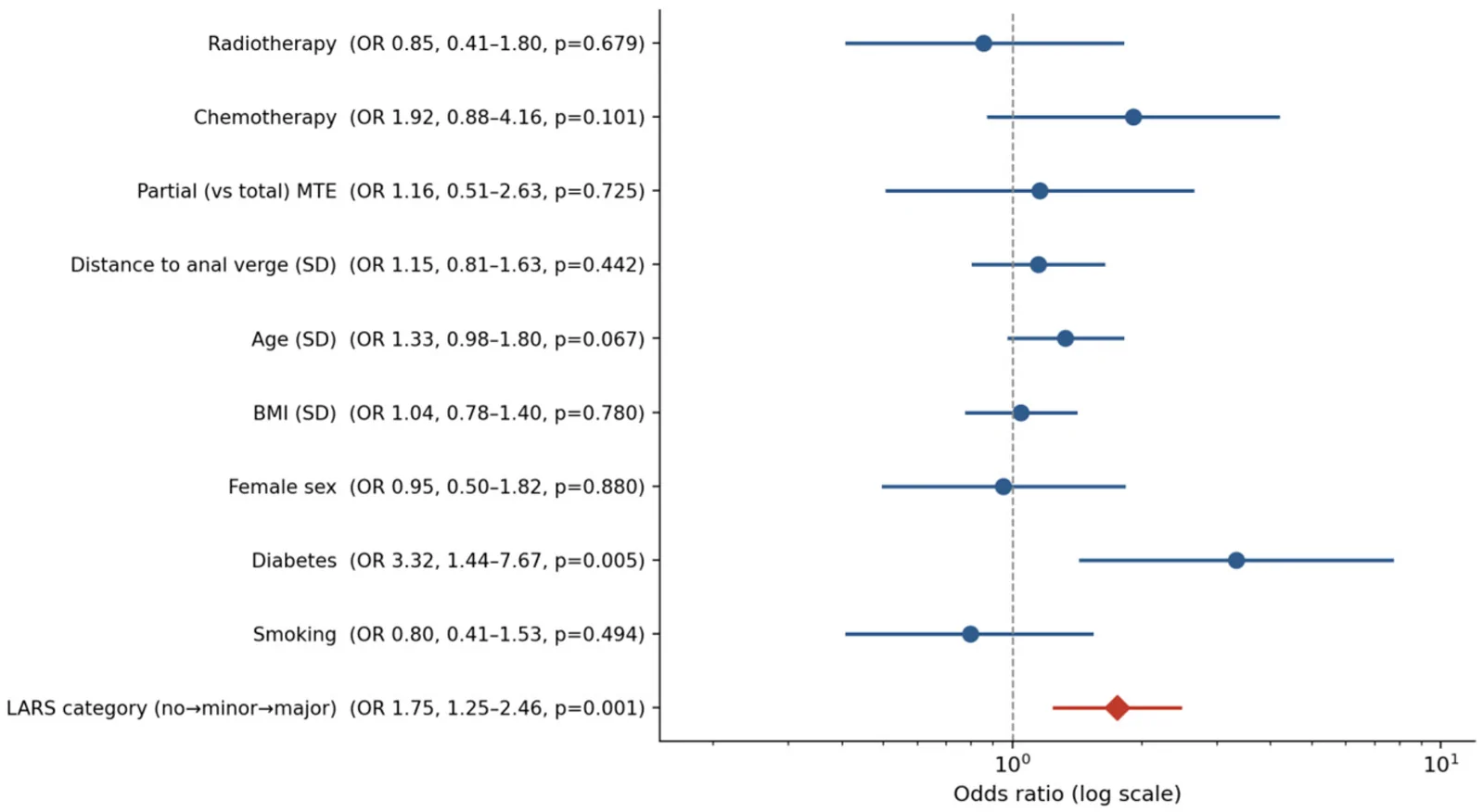
Forest plot of the primary Model C estimates for IPSS severity. Odds ratios are shown with 95% confidence intervals.

Model C added the LARS category to the Model B covariates and was fitted in the full cohort (n = 190). Inclusion of LARS significantly improved model fit compared with Model B (likelihood-ratio χ^2^ = 10.85, df = 1, *p* = 0.0010). Each one-category increase in LARS severity was associated with greater IPSS severity (OR 1.75, 95% CI 1.25–2.46, *p* = 0.0012). Diabetes also remained associated with IPSS severity (OR 3.32, 95% CI 1.44–7.67, *p* = 0.0049), while no other covariate was statistically significant (Table 5; Figure 7).

### 3.5. Sensitivity and Model-Diagnostic Analyses

#### 3.5.1. Alternative Specifications

**Figure 8:**
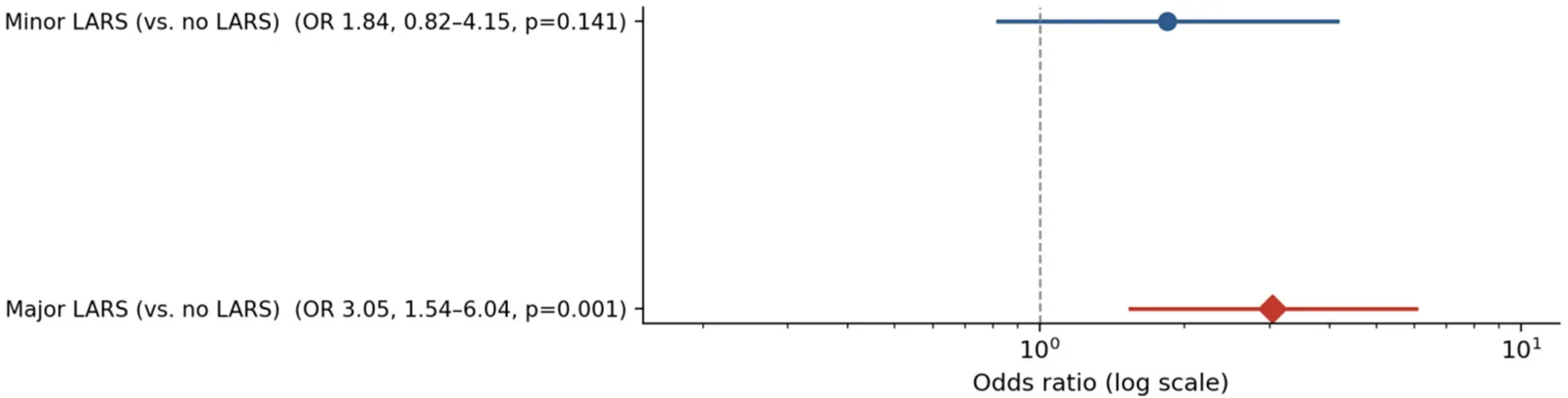
Forest plot of the sensitivity analysis with minor and major LARS entered as separate indicators. No LARS was the reference category.

The association remained similar when tumour height was represented categorically (OR 1.80, 95% CI 1.27–2.54, *p* = 0.0009) and when minor and major LARS were entered separately. Compared with no LARS, major LARS was associated with greater IPSS severity (OR 3.05, 95% CI 1.54–6.04, *p* = 0.0014), whereas the estimate for minor LARS was imprecise (OR 1.84, 95% CI 0.82–4.15, *p* = 0.141) (Table 6; Figure 8).

#### 3.5.2. Follow-Up and Protective Ileostomy

Adjustment for protective ileostomy produced a similar LARS estimate (OR 1.64, 95% CI 1.16–2.33, *p* = 0.0053; n = 182). Follow-up duration was not associated with IPSS severity, and adjustment for follow-up did not materially alter the LARS estimate. The association also remained present after excluding patients assessed within 3 months (OR 1.60, *p* = 0.0072; n = 176) or 6 months (OR 1.79, *p* = 0.0016; n = 168) of restoration of bowel continuity (Table 7).

#### 3.5.3. Other Robustness Analyses

Continuous-score regression showed a similar association between LARS score and IPSS (β = 1.49, 95% CI 0.66–2.32, *p* = 0.0005). There was no statistically significant LARS-by-sex interaction (*p* = 0.133), although sex-stratified estimates were imprecise (Table 8). The association persisted after combining moderate and severe IPSS categories (OR 1.66, 95% CI 1.19–2.33, *p* = 0.0030). Bootstrap resampling produced a median OR of 1.81 with a 95% percentile-based confidence interval of 1.26–2.65. No evidence of complete or quasi-complete separation was identified at the severe-IPSS threshold (largest predictor coefficient 1.32, SE 1.40; predicted probabilities 0.0004–0.273); LARS category itself showed no evidence of separation (OR 1.67, 95% CI 0.55–5.07, *p* = 0.366).

#### 3.5.4. Additional Clinical and Treatment Sensitivity Analyses

Pre-existing urinary symptoms were documented in 29 patients (15.3%). After additional adjustment for this variable, the association between LARS and IPSS severity remained essentially unchanged (OR 1.77, 95% CI 1.26–2.49, *p* = 0.0010). Pre-existing urinary symptoms were not associated with postoperative IPSS severity after adjustment (OR 0.73, 95% CI 0.31–1.73, *p* = 0.480).

Anastomotic leakage/pelvic sepsis occurred in 18 of 190 patients (9.5%). In the categorical tumour-position sensitivity model, additional adjustment for leakage/pelvic sepsis left the LARS estimate essentially unchanged (OR 1.79, 95% CI 1.27–2.54, *p* = 0.0009); leakage/pelvic sepsis was not associated with IPSS severity (OR 1.30, 95% CI 0.49–3.44, *p* = 0.597). Anastomotic configuration was available in 182 patients and likewise did not materially affect the LARS association (OR 1.80, 95% CI 1.25–2.58, *p* = 0.0015); configuration itself was not associated with IPSS severity (OR 1.10, 95% CI 0.58–2.08, *p* = 0.770). Rectal stump length was available in 110 patients; in this smaller subset, the LARS estimate remained directionally similar but was less precise (OR 1.53, 95% CI 0.97–2.41, *p* = 0.066), and stump length was not associated with IPSS severity (OR 0.72 per SD increase, 95% CI 0.45–1.17, *p* = 0.185).

Postoperative medication for digestive symptoms was recorded in 45 patients (23.7%) and medication for urinary symptoms in 42 patients (22.1%). Adjustment for digestive-symptom medication did not alter the LARS estimate (OR 1.75, 95% CI 1.25–2.46, *p* = 0.0012), and adjustment for urinary-symptom medication produced a similarly stable estimate (OR 1.76, 95% CI 1.25–2.47, *p* = 0.0012). When both medication variables were included simultaneously, the LARS association remained unchanged (OR 1.75, 95% CI 1.24–2.46, *p* = 0.0013).

#### 3.5.5. Model Diagnostics

The proportional-odds assumption was assessed using the Brant test. No evidence of violation was identified for Model A (χ^2^ = 2.61, df = 9, *p* = 0.978), Model B (χ^2^ = 8.96, df = 18, *p* = 0.961), or Model C (χ^2^ = 10.39, df = 20, *p* = 0.961). A predictor-specific Brant test for LARS category alone in Model C likewise showed no evidence of violation (χ^2^ = 0.95, df = 2, *p* = 0.621), indicating that the proportional-odds assumption held specifically for the predictor of primary interest. Variance inflation factors ranged from 1.06 (BMI) to 1.70 (radiotherapy), indicating no evidence of relevant multicollinearity (Supplementary Table S2).

## 4. Discussion

The principal finding of this study was that greater postoperative LARS severity was associated with greater contemporaneously measured lower urinary tract symptom burden after adjustment for the available demographic, clinical, anatomical, and treatment-related variables. In the primary full-cohort model, each increase from no to minor or from minor to major LARS was associated with a 75% increase in the odds of belonging to a higher IPSS category. Inclusion of LARS significantly improved the fit of a model that already incorporated neoadjuvant radiotherapy, chemotherapy, extent of mesorectal excision, MRI-measured distance from the tumour to the anal verge, age, sex, body mass index, diabetes, and smoking.

The association was materially similar across a broad series of correlated sensitivity analyses: alternative LARS coding, categorical rather than continuous tumour height, adjustment for follow-up duration and protective ileostomy, sex-stratified models, consolidation of the sparse severe-IPSS category, continuous-score regression, and bootstrap resampling, which closely reproduced the primary odds ratio with no evident separation at the severe-IPSS threshold. Additional adjustment for recorded pre-existing urinary symptoms, anastomotic leakage/pelvic sepsis, anastomotic configuration, and postoperative digestive- or urinary-symptom medication likewise left the LARS estimate materially unchanged. In the smaller subgroup with available rectal stump length, the estimate remained directionally similar but less precise. When LARS was modelled as separate indicators, patients with major LARS had approximately threefold higher odds of a more severe IPSS category than those without LARS; the minor-LARS estimate was smaller, imprecise, and based on only 30 patients, and should not be read as evidence of a strict threshold between minor and major LARS. The unadjusted correlation and adjusted LARS estimate were numerically larger in women, but the LARS-by-sex interaction was not significant, so the study does not establish that the association differs by sex. The main association was therefore not materially dependent on a single anatomical definition, coding choice, follow-up restriction, covariate specification, outcome categorisation, or analytic approach.

Although the correlation between the continuous LARS score and IPSS was modest, its clinical relevance should not be judged solely by the magnitude of the correlation coefficient. The two instruments assess distinct symptom complexes, and a strong linear relationship would not necessarily be expected even if bowel and urinary dysfunction shared some determinants. More importantly, moderate-to-severe LUTS were present in 58.5% of patients with major LARS, compared with 38.7% of those with no or minor LARS. The continuous-score association therefore corresponded to a clinically meaningful concentration of substantial urinary symptom burden among patients with the most severe bowel dysfunction. The close agreement between Pearson and Spearman coefficients also suggests that the finding was not driven by applying a parametric correlation measure to bounded, discrete questionnaire scores.

Few studies have directly evaluated bowel and urinary symptom severity together after restorative rectal cancer surgery. Kim et al. reported that major LARS and moderate-to-severe LUTS were both common after sphincter-preserving rectal cancer surgery and that greater symptom severity in either domain was associated with poorer quality of life [23]. Their study established the clinical relevance of concurrent bowel and urinary assessment but did not principally determine whether the two symptom domains remained associated after adjustment for shared clinical and treatment-related factors. This distinction is important because prospective studies with baseline assessment have demonstrated that urinary symptom trajectories after rectal cancer surgery are heterogeneous and that postoperative scores cannot automatically be interpreted as newly treatment-induced dysfunction [14,15]. Earlier evidence from the Dutch TME trial showed that newly developed faecal and urinary incontinence were associated with rectal cancer treatment [19,20]. Studies in women have likewise demonstrated that bowel, urinary, and sexual dysfunction may coexist after rectal cancer resection [21,22]. The present study extends this literature by showing that ordered LARS and IPSS severity remained associated after adjustment for several measured exposures that could plausibly contribute to both conditions.

The plausibility of shared pelvic neural injury is supported by studies of pelvic autonomic nerve preservation and intraoperative neuromonitoring, which have evaluated bowel, urinary, and sexual outcomes as interconnected functional consequences of pelvic dissection [27,28]. Nevertheless, the present findings do not demonstrate that rectal cancer treatment caused the urinary symptoms, that LARS and LUTS developed simultaneously, or that a single pelvic nerve lesion caused both symptom complexes. LARS is a heterogeneous syndrome arising from loss of rectal reservoir capacity, altered neorectal compliance and motility, disruption of anorectal sensation and reflexes, sphincter dysfunction, radiation-related injury, temporary diversion, and altered pelvic floor coordination [2,3,9]. IPSS similarly measures a broad mixture of storage and voiding symptoms and does not identify their anatomical cause [24,25]. Postoperative LUTS may reflect pelvic autonomic injury, but they may also arise from benign prostatic enlargement, overactive bladder, diabetes, ageing, medication use, infection, or pre-existing urological disease [16,25,26]. The observed association may therefore reflect several partially overlapping mechanisms, residual confounding, pre-existing urinary disease, or a broader susceptibility to symptom burden rather than one uniform postoperative bowel–bladder syndrome.

A further possible explanation is that some patients have a broader vulnerability to multiple functional symptoms or report greater burden across patient-reported instruments. Rectal cancer survivorship commonly involves concurrent bowel, urinary, sexual, psychological, and social consequences, and these domains may interact even when their biological mechanisms are not identical [29,30]. Symptoms in one domain can amplify the perceived burden of another through sleep disruption, avoidance of travel or social activity, anxiety about continence, and reduced confidence in leaving home. Long-term survivorship studies have shown that bowel dysfunction is associated with restrictions in daily participation and quality of life, reinforcing the importance of interpreting symptom scores within a broader functional context rather than as isolated organ-specific measurements [29,31,32,33]. Multidisciplinary survivorship reviews similarly emphasise that chronic toxicities after rectal cancer treatment often involve several organ systems and require integrated rather than fragmented follow-up.

The item-level analysis did not identify one dominant shared symptom pathway. Four weak nominal associations were observed, involving urgency-, frequency-, intermittency-, incomplete-emptying-, and nocturia-related items, but none remained significant after correction for 35 comparisons. This diffuse pattern suggests that the association between total LARS and IPSSs was not driven by a single pair of analogous questions. Composite instruments may capture the cumulative effect of several small relationships more reliably than isolated items, particularly when bowel and urinary dysfunction arise through heterogeneous combinations of denervation, altered sensation, pelvic floor discoordination, reservoir dysfunction, comorbidity, and individual symptom susceptibility. The item-level findings should therefore remain exploratory. Future mechanistic studies should prespecify clinically coherent symptom domains and combine questionnaires with objective measures such as uroflowmetry, postvoid residual volume, urodynamics, anorectal manometry, continence testing, and pelvic floor imaging.

Diabetes was the other consistent correlate of IPSS severity. Patients with diabetes had more than threefold higher adjusted odds of belonging to a more severe urinary symptom category. Diabetic bladder dysfunction may include reduced bladder sensation, impaired detrusor contractility, increased residual urine, urgency, frequency, nocturia, or mixed storage and voiding symptoms [26]. Its phenotype varies according to diabetes duration, glycaemic control, autonomic neuropathy, age, and concomitant urological disease. This finding supports interpretation of postoperative IPSS as a nonspecific measure of urinary symptom burden rather than a direct marker of surgical autonomic injury. Clinicians evaluating LUTS after rectal cancer surgery should therefore consider metabolic and pre-existing urological contributors rather than automatically attributing all symptoms to pelvic dissection.

Neither neoadjuvant radiotherapy nor the recorded extent of mesorectal excision was associated with IPSS severity after multivariable adjustment. By contrast, a recent meta-analysis found that neoadjuvant therapy was associated with increased anorectal dysfunction and a modestly increased risk of urinary incontinence, although the authors emphasised the influence of tumour stage, operative approach, and other confounding factors [11]. This differs from the Dutch TME analysis, in which urinary dysfunction was attributed predominantly to surgery rather than radiotherapy [19]. Several explanations are possible. Total versus partial mesorectal excision is an incomplete proxy for the technical difficulty and neural consequences of pelvic dissection and does not capture mesorectal plane quality, direct identification of autonomic nerves, traction, thermal injury, bleeding, conversion, pelvic dimensions, anastomotic height, or operator-related factors. In addition, radiotherapy, chemotherapy, tumour level, stage, and surgical extent are clinically interrelated rather than randomly assigned exposures. IPSS may also fail to detect some forms of postoperative bladder impairment as sensitively as catheter dependence, postvoid residual measurement, or formal urodynamics.

Recent reviews and meta-analyses demonstrate substantial heterogeneity in both bowel and urinary outcomes after rectal cancer surgery, reflecting differences in patient selection, operative approach, neoadjuvant treatment, outcome instruments, baseline assessment, and follow-up duration [5,11,13,34,35,36,37]. They have not shown consistent long-term superiority of one operative approach across urinary, sexual, bowel, and quality-of-life outcomes, although selected analyses suggest possible differences in early recovery [35,36]. Accordingly, the absence of an adjusted radiotherapy or operative effect in the present cohort should not be interpreted as evidence that treatment technique is unimportant. Rather, it illustrates the limitations of using broad treatment categories to represent the specific neural and functional consequences of pelvic surgery.

Tumour height is a recognised predictor of LARS and reflects the level and technical complexity of pelvic dissection [4,12]. In the categorical sensitivity analysis, lower rectal tumour location was associated with greater LARS severity, supporting the anatomical classification’s validity, but substituting categorical position for continuous distance did not materially change the adjusted LARS–IPSS association. Additional adjustment for protective ileostomy produced only modest attenuation of the LARS estimate, and ileostomy itself was associated with LARS but not IPSS severity; both remain broad proxies that do not capture nerve exposure, dissection quality, or anastomotic level. Follow-up duration from restoration of bowel continuity likewise did not alter the LARS estimate, and excluding patients assessed within 3 or 6 months did not eliminate the association, reducing concern that transient early symptoms drove the finding. This does not establish that symptom burden is stable over time: LARS may improve, persist, or fluctuate, with most improvement reported during the first postoperative months and substantial symptoms often persisting thereafter [6,7,38], while urinary function may follow a different trajectory [14,15]. Cross-sectional assessment cannot determine whether bowel and urinary symptoms emerged simultaneously or which, if either, preceded the other.

Recent patient-centred studies further indicate that bowel dysfunction affects daily activities, work, social participation, and confidence and that patients value proactive, structured follow-up rather than having to initiate discussion of functional symptoms themselves [39,40]. In clinical follow-up, major LARS may serve as a practical trigger for brief assessment of coexisting urinary urgency, frequency, nocturia, impaired emptying, weak stream, retention, and incontinence. Patients reporting bothersome, progressive, or otherwise clinically significant LUTS may then undergo medication review, assessment for metabolic or pre-existing urological contributors, and targeted urological evaluation when clinically indicated. This approach should be regarded as symptom-oriented case finding rather than evidence that LARS causes urinary dysfunction or that treatment of LARS will necessarily improve LUTS.

Future multicentre studies should incorporate preoperative and repeated postoperative bowel and urinary assessments, objective anorectal and urological testing, and detailed surgical and comorbidity data to clarify temporal relationships and externally validate these findings.

Several features of this study strengthen the interpretation of its findings. Bowel and urinary symptoms were assessed concurrently using structured administration of validated severity instruments, thereby avoiding temporal mismatch between functional domains. Both outcomes were analysed using their full ordered categories, and the primary proportional-odds model adjusted for clinically relevant demographic, anatomical, oncological, and treatment-related variables while directly testing the additional contribution of LARS to IPSS severity. The stability of the LARS estimate was examined across multiple correlated robustness analyses, including alternative coding of tumour height and LARS, adjustment for follow-up duration and protective ileostomy, sex-specific analyses, consolidation of the sparse severe-IPSS category, continuous-score modelling, and bootstrap resampling. Multiplicity in the exploratory item-level analysis was addressed explicitly, and no unsupported symptom-specific mechanism was inferred. This study has several limitations. Its retrospective, single-centre design and cross-sectional postoperative assessment precluded evaluation of symptom onset, temporal direction, or treatment causality. Standardised preoperative IPSS measurements were not available; although a documented history of pre-existing urinary symptoms was examined in sensitivity analysis, residual confounding from baseline urinary dysfunction remains possible.

IPSS is a general LUTS instrument rather than a measure specific to pelvic autonomic injury and was originally developed for men, although it has also been used in women and mixed-sex populations. A female-specific or sex-neutral validated LUTS instrument was not available in this dataset; therefore, the content validity of IPSS in women remains a limitation that is distinct from the statistical question of whether the LARS–IPSS association differs by sex. The severe-IPSS category included only five patients, limiting threshold-specific precision despite reassuring results from category consolidation, bootstrap resampling, and separation assessment. Several potentially relevant confounders were not systematically available, including benign prostatic enlargement, overactive bladder, medications specifically capable of causing voiding dysfunction, diabetes duration and control, mesorectal plane quality, and objective measures of autonomic nerve preservation. Educational level and structured bladder rehabilitation or bladder-training programmes were also not systematically recorded. All patients received routine lifestyle advice, but the intensity of and adherence to such advice were not quantified. Although postoperative digestive- and urinary-symptom medication was recorded and did not materially alter the LARS–IPSS association in sensitivity analyses, treatment was likely initiated in response to symptom burden and therefore cannot be interpreted as a baseline confounder. Rectal stump length was available in only a restricted subset, limiting precision in that analysis. Residual confounding therefore remains possible. The item-level analysis was exploratory; none of the nominal associations survived multiplicity correction, and telephone administration may have introduced recall or interviewer effects. The substantial exclusion of patients, predominantly because of non-response or declined participation, may have introduced response-selection bias, while exclusion of patients who died before functional assessment may also have contributed to survivorship bias. Baseline characteristics were not available for the excluded patients; therefore, we could not compare included and excluded patients to assess the direction or magnitude of potential selection bias. Missing data were limited to secondary variables and were handled using available-case analyses without imputation; however, the possibility of bias related to non-random missingness cannot be excluded. Finally, the study was not prospectively registered and had no time-stamped statistical analysis plan, so the findings should be considered hypothesis-supporting and require confirmation in prospective multicentre cohorts.

## 5. Conclusions

Greater postoperative LARS severity was associated with greater contemporaneously measured IPSS severity after adjustment for the available demographic, clinical, anatomical, and treatment-related variables. The association remained materially similar across alternative representations of tumour position and LARS severity, adjustment for follow-up duration and protective ileostomy, sex-specific analyses, consolidation of the sparse severe-IPSS category, continuous-score modelling, and bootstrap assessment.

These findings support the coexistence of bowel and lower urinary tract symptom burden after restorative rectal cancer surgery but do not establish temporal direction, treatment causality, or a shared postoperative mechanism. Concurrent assessment of bowel and urinary symptoms may help identify patients with broader pelvic functional needs during survivorship follow-up. Prospective longitudinal studies incorporating preoperative assessment, repeated postoperative measurements, and objective anorectal and urological testing are needed to clarify symptom trajectories and underlying mechanisms.

## Figures and Tables

**Table 1:** Demographic, clinical, treatment, and functional characteristics of the study cohort.

Characteristic	Value
Age, years (mean ± SD)	64.8 ± 9.9
Female sex, n (%)	87 (45.8)
BMI, kg/m^2^ (mean ± SD)	27.7 ± 4.6
Diabetes, n (%)	31 (16.3)
Smoking history, n (%)	83 (43.7)
Neoadjuvant radiotherapy, n (%)	114 (60.0)
Chemotherapy, n (%)	144 (75.8)
Total mesorectal excision, n (%)	141 (74.2)
Partial mesorectal excision, n (%)	49 (25.8)
Distance to anal verge, mm (mean ± SD)	97.2 ± 39.1 (median 90.0, IQR 70.0–125.0)
Follow-up from restoration of bowel continuity, months, median [IQR], n = 181	26.6 [13.5–63.9], range 0.8–131.0
Protective ileostomy, n/N (%)	116/182 (63.7)
LARS—no LARS, n (%)	107 (56.3)
LARS—minor LARS, n (%)	30 (15.8)
LARS—major LARS, n (%)	53 (27.9)
LARS score (mean ± SD)	19.3 ± 12.6
IPSS—none, n (%)	23 (12.1)
IPSS—mild, n (%)	83 (43.7)
IPSS—moderate, n (%)	79 (41.6)
IPSS—severe, n (%)	5 (2.6)
IPSS (mean ± SD)	7.5 ± 5.8

**Note:** Data are presented as mean ± standard deviation, median [interquartile range] and range, or number (percentage), as appropriate. Unless otherwise specified, n = 190.

**Table 2:** Spearman correlation coefficients between individual LARS and IPSS items (n = 175).

LARS Item	IPSS Q1 Emptying	IPSS Q2 Frequency	IPSS Q3 Intermit.	IPSS Q4 Urgency	IPSS Q5 Weak Stream	IPSS Q6 Straining	IPSS Q7 Nocturia
Q1 Flatus incontinence	0.134	0.079	0.058	0.061	0.132	0.081	0.049
Q2 Liquid incontinence	0.128	0.152 *	0.063	0.023	0.083	0.110	0.075
Q3 Frequency	0.122	0.137	0.095	0.138	0.038	0.127	0.043
Q4 Clustering	0.020	0.129	0.032	0.021	0.062	0.077	0.170 *
Q5 Urgency	0.149 *	0.089	0.173 *	0.098	0.102	0.079	0.112

**Note:** Values are Spearman correlation coefficients. Asterisks indicate nominal *p* < 0.05. None of the 35 item-level associations met the Bonferroni-corrected significance threshold of *p* < 0.0014.

**Table 3:** Model A: proportional-odds regression of LARS category on predefined demographic, clinical, anatomical, and treatment-related variables (n = 190).

Predictor	OR	95% CI	*p*
Radiotherapy	1.42	0.66–3.05	0.376
Chemotherapy	1.37	0.60–3.09	0.454
Partial (vs. total) mesorectal excision	0.83	0.36–1.94	0.668
Distance to anal verge (per SD)	0.82	0.58–1.18	0.290
Age (per SD)	0.85	0.61–1.17	0.308
BMI (per SD)	1.26	0.94–1.69	0.126
Female sex	1.00	0.51–1.95	0.994
Diabetes	0.92	0.43–2.01	0.840
Smoking	1.67	0.86–3.25	0.132

**Note:** Odds ratios for continuous variables are reported per one-standard-deviation increase.

**Table 4:** Model B: proportional-odds regression of IPSS category on predefined demographic, clinical, anatomical, and treatment-related variables (n = 190).

Predictor	OR	95% CI	*p*
Radiotherapy	0.96	0.46–1.98	0.905
Chemotherapy	1.99	0.92–4.28	0.080
Partial (vs. total) mesorectal excision	1.12	0.50–2.51	0.791
Distance to anal verge (per SD)	1.10	0.78–1.56	0.589
Age (per SD)	1.30	0.96–1.76	0.088
BMI (per SD)	1.10	0.83–1.47	0.501
Female sex	0.95	0.49–1.81	0.867
Diabetes	3.17	1.39–7.24	0.006
Smoking	0.88	0.46–1.67	0.696

**Note:** Odds ratios for continuous variables are reported per one-standard-deviation increase.

**Table 5:** Model C: primary proportional-odds regression of IPSS category on predefined demographic, clinical, anatomical, and treatment-related variables plus LARS category (n = 190).

Predictor	OR	95% CI	*p*
Radiotherapy	0.86	0.41–1.80	0.679
Chemotherapy	1.92	0.88–4.16	0.101
Partial (vs. total) mesorectal excision	1.16	0.51–2.63	0.725
Distance to anal verge (per SD)	1.15	0.81–1.63	0.442
Age (per SD)	1.33	0.98–1.80	0.067
BMI (per SD)	1.04	0.78–1.40	0.780
Female sex	0.95	0.50–1.82	0.880
Diabetes	3.32	1.44–7.67	0.0049
Smoking	0.80	0.41–1.53	0.494
LARS category (no → minor → major)	1.75	1.25–2.46	0.0012

**Note:** Odds ratios for continuous variables are reported per one-standard-deviation increase. The LARS estimate represents the change in the odds of belonging to a higher IPSS category for each one-category increase from no to minor or from minor to major LARS.

**Table 6:** Sensitivity analysis with LARS represented by separate indicator variables in the primary IPSS model (n = 190).

Predictor	OR	95% CI	*p*
Minor LARS (vs. no LARS)	1.84	0.82–4.15	0.141
Major LARS (vs. no LARS)	3.05	1.54–6.04	0.0014

**Note:** No LARS was the reference category. The model was adjusted for neoadjuvant radiotherapy, chemotherapy, extent of mesorectal excision, MRI-measured distance from the tumour to the anal verge, age, sex, body mass index, diabetes, and smoking.

**Table 7:** Sensitivity analysis with additional adjustment for protective ileostomy status (n = 182). (A). Extension of Model A: factors associated with LARS severity after inclusion of protective ileostomy status. (B). Extension of Model C: factors associated with IPSS severity after inclusion of protective ileostomy status.

**(A)**			
**Predictor**	**OR**	**95% CI**	***p***
Radiotherapy	1.00	0.43–2.31	0.999
Chemotherapy	1.38	0.60–3.21	0.451
Partial (vs. total) mesorectal excision	1.19	0.49–2.92	0.703
Distance to anal verge (per SD)	0.84	0.58–1.22	0.351
Age (per SD)	0.80	0.58–1.12	0.195
BMI (per SD)	1.31	0.96–1.79	0.093
Female sex	1.02	0.51–2.04	0.950
Diabetes	1.10	0.50–2.46	0.809
Smoking	1.88	0.94–3.74	0.073
**Protective ileostomy**	**2.86**	**1.25–6.56**	**0.013**
**(B)**			
**Predictor**	**OR**	**95% CI**	***p***
Radiotherapy	0.78	0.36–1.68	0.518
Chemotherapy	1.88	0.87–4.07	0.109
Partial (vs. total) mesorectal excision	1.33	0.56–3.13	0.515
Distance to anal verge (per SD)	1.14	0.80–1.63	0.455
Age (per SD)	1.30	0.95–1.77	0.102
BMI (per SD)	1.06	0.79–1.44	0.690
Female sex	0.91	0.47–1.76	0.781
Diabetes	3.47	1.48–8.11	0.0041
Smoking	0.82	0.43–1.59	0.563
Protective ileostomy	1.40	0.66–2.98	0.383
**LARS category (no → minor → major)**	**1.64**	**1.16–2.33**	**0.0053**

**Note:** Panel A used LARS category as the outcome. Panel B used IPSS category as the outcome and included LARS category as an ordered predictor. In Panel B, the model including LARS had a log-likelihood of −184.67, compared with −188.67 for the otherwise identical model without LARS (likelihood-ratio χ^2^ = 8.00, df = 1, *p* = 0.0047). Odds ratios for continuous variables are reported per one-standard-deviation increase. The LARS estimate represents the change in the odds of belonging to a higher IPSS category for each one-category increase from no to minor or from minor to major LARS.

**Table 8:** Sex-stratified descriptive characteristics and LARS–IPSS correlations (n = 190).

Characteristic	Men (n = 103)	Women (n = 87)	*p*
LARS score (mean ± SD)	19.7 ± 13.0	18.8 ± 12.3	0.615
IPSS (mean ± SD)	7.8 ± 5.9	7.1 ± 5.7	0.387
No LARS, n (%)	53 (51.5)	54 (62.1)	–
Minor LARS, n (%)	20 (19.4)	10 (11.5)	–
Major LARS, n (%)	30 (29.1)	23 (26.4)	–
No IPSS, n (%)	12 (11.7)	11 (12.6)	–
Mild IPSS, n (%)	44 (42.7)	39 (44.8)	–
Moderate IPSS, n (%)	44 (42.7)	35 (40.2)	–
Severe IPSS, n (%)	3 (2.9)	2 (2.3)	–
Pearson r (LARS score × IPSS)	0.136 (*p* = 0.171)	0.336 (*p* = 0.0015)	–

**Note:** Continuous scores are presented as mean ± standard deviation, and categorical variables as number (percentage). Pearson correlation coefficients were calculated separately in men and women. Between-sex *p*-values for continuous variables were obtained using Student’s *t*-test. Category distributions are presented descriptively.

## Data Availability

The original contributions presented in this study are included in the article/Supplementary Materials. Further inquiries can be directed to the corresponding author.

## Supplementary Materials

The following supporting information can be downloaded at https://www.mdpi.com/article/10.3390/medsci14060682/s1: Figure S1: Analytical flowchart and study subsets; Table S1: STROBE checklist for cohort studies; Table S2: Variance inflation factors, Model C (n = 190).

## Abbreviations

The following abbreviations are used in this manuscript:

BMI	Body mass index
CI	Confidence interval
IPSS	International Prostate Symptom Score
IQR	Interquartile range
IRO	Regional Institute of Oncology
LARS	Low anterior resection syndrome
LUTS	Lower urinary tract symptoms
MRI	Magnetic resonance imaging
OR	Odds ratio
SD	Standard deviation
SE	Standard error
STROBE	Strengthening the Reporting of Observational Studies in Epidemiology
TME	Total mesorectal excision
