# Supplementary material for: Association Between Low Anterior Resection Syndrome and Lower Urinary Tract Symptom Severity After Restorative Rectal Cancer Surgery
Source: Med Sci (Basel). 2026 Oct 19;14(6):682. doi: 10.3390/medsci14060682 (PMC13706910; doi:10.3390/medsci14060682)
Supplement: Supplementary file 1 [file medsci-14-00682-s001.zip › medsci-4625022-supplementary.pdf]

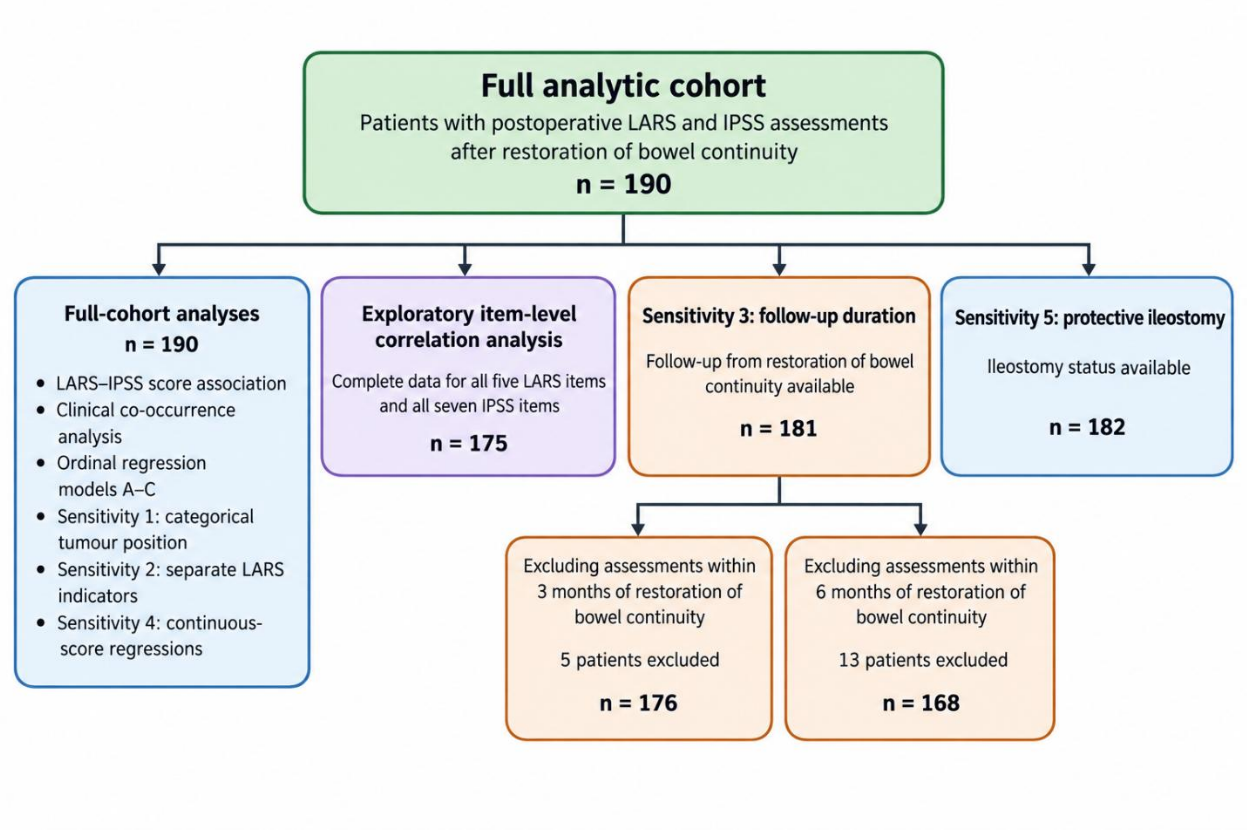

Supplementary Figure S1. Analytical flowchart and study subsets. All 190 patients in the full analytic cohort contributed to the LARS–IPSS score association, clinical co-occurrence analysis, ordinal regression models A–C, sensitivity analyses using categorical tumour position and separate LARS indicators, and continuous-score regression analyses. Complete responses for all five LARS and seven IPSS items were available for 175 patients. Follow-up from restoration of bowel continuity was available for 181 patients; separate robustness analyses retained 176 patients after excluding assessments within 3 months and 168 patients after excluding assessments within 6 months. Protective ileostomy status was available for 182 patients, who contributed to the additional complete-case sensitivity analysis.

Supplementary Table S1. STROBE checklist for cohort studies

STROBE item	Reporting requirement	Where addressed	Updated status
**1a. Title/design**	State study design using a commonly understood term.	Abstract; Section 2.1	**Complete.** The abstract identifies a retrospective single-centre cohort study.
**1b. Abstract**	Provide a balanced summary of methods and findings.	Abstract	**Complete.**
**2. Background/rationale**	Explain scientific background and rationale.	Introduction	**Complete.**
**3. Objectives**	State specific objectives/hypotheses.	Final paragraph of Introduction	**Complete.**
**4. Study design**	Present key design elements early.	Section 2.1	**Complete.** The manuscript states that the study was reported in accordance with the STROBE statement.
**5. Setting**	Describe setting, location, and relevant dates.	Sections 2.1–2.3	**Complete.**
**6a. Participants**	Give eligibility criteria and participant selection.	Section 2.2; Figure 1	**Complete.** Exclusion criteria counts: stoma 15, death 7, non-response/refusal 172, unreliable responses 9, non-operative/watch-and-wait 11.
**6b. Matching**	Describe matching if used.	—	**Not applicable.**
**7. Variables**	Define outcomes, exposures, predictors, confounders, effect modifiers.	Sections 2.3–2.5	**Complete.**
**8. Data sources/measurement**	Describe sources and measurement methods.	Sections 2.3–2.4	**Complete.** Postoperative bowel and urinary symptoms were assessed using the five-item LARS score and seven-item IPSS during the same structured telephone interview.
**9. Bias**	Describe efforts to address bias.	Methods; sensitivity analyses; Limitations	**Complete.** Selection bias, survivorship bias, residual confounding, and measurement limitations are acknowledged.
**10. Study size**	Explain how study size was determined.	Section 2.2	**Complete:** "No a priori sample-size calculation was performed; the study included all eligible patients with the

STROBE item	Reporting requirement	Where addressed	Updated status
			required postoperative functional assessments available during the study period.”
**11. Quantitative variables**	Explain handling/categorisation of quantitative variables.	Sections 2.3, 2.4, 2.6	**Complete.**
**12a. Statistical methods**	Describe statistical methods and confounder control.	Section 2.6	**Complete.**
**12b. Subgroups/interactions**	Describe subgroup and interaction analyses.	Section 2.6; Results 3.5.3	**Complete.**
**12c. Missing data**	Explain how missing data were handled.	Section 2.6	**Complete**. Analyses involving variables with missing data used available complete cases; no imputation was performed.
**12d. Loss to follow-up/attrition**	Explain how attrition was handled.	Section 2.2; Limitations	**Complete with limitation acknowledged.**
**12e. Sensitivity analyses**	Describe sensitivity analyses.	Section 2.6	**Complete.**
**13a. Participant flow**	Report numbers at each stage.	Figure 1; Section 2.2	**Complete.** 404 screened, 214 excluded with reason-specific counts, 190 analysed.
**13b. Reasons for non-participation**	Give reasons for exclusion/non-participation.	Section 2.2; Figure 1	**Complete.**
**13c. Flow diagram**	Consider use of a flow diagram.	Figure 1; Supplementary Figure S1	**Complete.**
**14a. Descriptive data**	Report participant characteristics.	Table 1	**Complete.**
**14b. Missing data by variable**	State numbers with missing data.	Section 2.2; Table 1	**Complete.** n=175 item-level, n=181 follow-up, n=182 ileostomy are identified.
**14c. Follow-up time**	Summarise follow-up.	Section 3.1; Table 1	**Complete.**
**15. Outcome data**	Report outcome measures/events.	Section 3.1; Table 1	**Complete.**
**16a. Main results**	Report unadjusted/adjusted estimates and precision.	Sections 3.2–3.4	**Complete.**

STROBE item	Reporting requirement	Where addressed	Updated status
**16b. Category boundaries**	Report cut-points for categorised continuous variables.	Sections 2.3–2.4	**Complete.**
**16c. Absolute measures**	Give meaningful absolute risks/differences when useful.	Section 3.2	**Complete.** The manuscript reports 58.5% vs 38.7% and a 19.8 percentage-point difference.
**17. Other analyses**	Report subgroup, interaction, and sensitivity analyses.	Section 3.5	**Complete.**
**18. Key results**	Summarise findings against objectives.	Opening Discussion	**Complete.**
**19. Limitations**	Discuss limitations and possible bias.	Final Discussion paragraph	**Complete.** Limitations include the mixed-sex use of IPSS and its content-validity limitations in women, response-selection and survivorship bias, inability to compare included and excluded patients, residual confounding, and possible bias from non-random missingness.
**20. Interpretation**	Give cautious overall interpretation.	Discussion	**Complete.**
**21. Generalisability**	Discuss external validity.	Limitations	**Complete with acknowledged limitation.** Baseline characteristics were unavailable for excluded patients, precluding comparison of included and excluded groups and limiting assessment of external validity and selection bias.
**22. Funding**	State funding and funder role.	End matter	**Complete.** This research received no external funding

Supplementary Table S2. Variance inflation factors, Model C (n=190).

Predictor	VIF
Radiotherapy	1.70
Distance to anal verge	1.59
Partial (vs. total) mesorectal excision	1.56
Chemotherapy	1.40
Female sex	1.40
Smoking	1.38
Age	1.21
LARS category	1.09
Diabetes	1.07
BMI	1.06
